# Soda consumption and the risk of metabolic syndrome in Korean adults during the period surrounding the COVID-19 pandemic

**DOI:** 10.1017/S0007114525106168

**Published:** 2026-04-14

**Authors:** Sehee Wi, Youjin Je

**Affiliations:** Department of Food and Nutrition, Kyung Hee University, Seoul 02447, South Korea

**Keywords:** Soda consumption, Metabolic syndrome, Sex difference, COVID-19, KNHANES

## Abstract

There is limited research on the association between soda consumption and the risk of metabolic syndrome (MetS), particularly during the COVID-19 pandemic. This study investigated the relationship between soda consumption and MetS in Korean adults, stratified by sex, and compared differences before and after the pandemic using data from the Korea National Health and Nutrition Examination Survey (2017–2021). A total of 13 051 adults aged 19–64 years were included. Soda consumption was assessed using 24-h recall and categorised into five groups (non-drinkers and four quartiles). Multivariable logistic regression models were used to estimate OR and 95 % CI for MetS and its components. After adjusting for multiple covariates, no significant association was found between soda consumption and MetS overall. However, adults in the highest quartile of soda consumption (≥ 373 g/d) had higher risks of abdominal obesity (*P*-trend = 0·006) and hypertriglyceridaemia (*P*-trend = 0·003), compared with non-drinkers. When analysed by gender, women in the highest quartile of soda consumption (≥ 315 g/d) had significantly higher risk of MetS (OR = 1·70; 95 % CI: 1·08, 2·68), and multiple MetS components, whereas no significant associations were obserbed in men. Post-pandemic analysis revealed a significant association between high soda consumption (≥ 416 g/d) and MetS (OR = 1·56; 95 % CI: 1·04, 2·34), which was NS in the pre-pandemic period (*P*–interaction = 0·031). These findings suggest that high soda consumption may increase the risk of MetS, particularly among Korean women.

Metabolic syndrome (MetS) is a complex condition characterised by metabolic irregularities such as abdominal obesity, high blood pressure, abnormal lipid levels, decreased HDL cholesterol and elevated blood glucose^(1)^. Diagnosis is confirmed when at least three of these criteria are present concurrently^(2)^. MetS significantly increases the risk of chronic kidney failure, stroke, diabetes and CVD, leading to higher mortality rates^(3,4)^. This condition poses a significant risk for developing type 2 diabetes and cardiovascular issues, as it is marked by a combination of factors such as insulin resistance. Globally, MetS is a prevalent health concern. In the USA, its prevalence increased from 32·5 % in 2011–2012 to 36·9 % in 2015–2016^(5,6)^. Similarly, the Asia-Pacific region has seen rising trends. In Korea, studies have documented an increase in MetS prevalence from 24·9 % in 1998 to 31·3 % in 2007^(7,8)^. Specifically, the prevalence among Korean men rose from 25·8 % to 40·0 % between 2001 and 2020, while it decreased among women from 28·2 % to 26·2 %^(9)^. These trends suggest gender-specific differences in MetS components and lifestyle changes. Sex and gender differences play a critical role in obesity and metabolic health, with men being more prone to visceral fat accumulation and earlier onset of cardiometabolic diseases, whereas women’s metabolic risk is shaped by hormonal fluctuations, reproductive factors and sociocultural influences on dietary preferences, such as food choices and beverage consumption patterns^(10)^.

Soda consumption has increased globally, and it is estimated that sugary drinks can raise daily caloric intake by up to 50 %^(11,12)^. In 2013, South Koreans consumed an average of 72·1 g of sugar daily, with a significant portion from beverages^(13)^. Between 2018 and 2022, average soda consumption in Korea increased from 42·8 g to 45·8 g per day^(14)^. There are several studies that have reported adverse associations between high sugar-sweetened beverage (SSB) intake, MetS and some of its components^(15–17)^. However, there is a lack of studies that examine the association of soda consumption with MetS and its components, especially during the COVID-19 pandemic, in Korean population. The pandemic has complicated the landscape, with potential cardiovascular complications and increased risk factors associated with MetS^(18,19)^. Moreover, emerging evidence suggests that the relationship between SSB, including soda, and metabolic risk factors may differ by sex, with stronger associations often observed in women than in men. This highlights the importance of investigating sex-specific differences to identify vulnerable populations and inform tailored public health strategies^(15)^.

Therefore, we aimed to assess the impact of soda consumption on MetS and its components in the general Korean adult population, stratified by sex, using data from the Korea National Health and Nutrition Examination Survey (KNHANES). In addition, we conducted a stratified analysis by the COVID-19 periods to compare whether the association between soda consumption and MetS differs before and after the COVID pandemic.

## Materials and methods

### Study population

**Figure 1.**
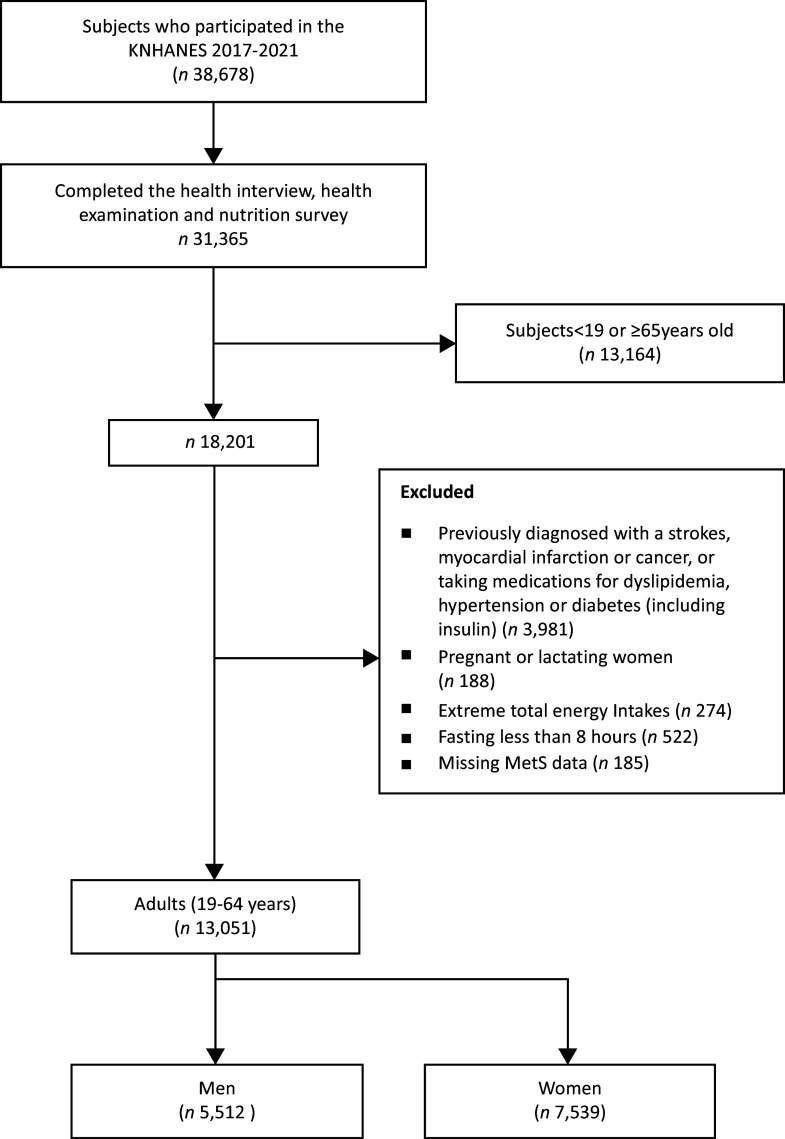
Figure 1 long description. Study participants were included in the study after the exclusion criteria. Note: The figure shows the selection process of the study population from the initial sample in the KNHANES 2017–2021 dataset. Participants were first filtered by age (excluding those under 19 or over 65 years). Further exclusions were made for individuals with a history of stroke, myocardial infarction or cancer, those taking medications for dyslipidemia, hypertension or diabetes, pregnant or lactating women, individuals with extreme total energy intakes (< 500 or > 5000 kcal/d), those who fasted for less than 8 h and those with missing MetS data. The final eligible sample consisted of 13 051 adults aged 19–64 years, divided into 5512 men and 7539 women.

Our study used data derived from the 7th period (2017–2018) and 8th period (2019–2021) of the KNHANES, administered by the Korea Centers for Disease Control and Prevention under the auspices of the Korean Ministry of Health and Welfare. The KNHANES employed a sophisticated sampling design, incorporating multistage, stratified, clustered and probability-based methodologies to capture a nationally representative cross-sectional survey. This survey consists of three principal components: a health examination, a health interview and a nutrition survey. Detailed descriptions of KNHANES methodologies have been earlier documented in literature^(20)^. Data were gathered from 38 678 participants in 2017 (*n* 8127), 2018 (*n* 7992), 2019 (*n* 8110), 2020 (*n* 7359) and 2021 (*n* 7090). Among them, 31 365 participants completed the health examination, health interview and nutrition survey. From this pool, participants were sequentially excluded based on the following criteria: individuals aged < 19 or ≥ 65 years (*n* 13 164); those self-reporting a history of myocardial infarction, stroke or cancer, or taking medications for hypertension, dyslipidemia or diabetes (*n* 3981); lactating or pregnant women (*n* 188); individuals with extreme total energy intakes (*n* 274); subjects who fasted less than 8 h (*n* 522); participants with missing data on MetS (*n* 185), consisting of a total of 13 051 participants (5512 men, 7539 women). The flow chart in Figure 1 illustrates the inclusion criteria for the final eligible study population. Prior to the survey, each participant was provided with informed consent, and the KNHANES dataset received formal ethics approval from the Institutional Review Board of the Korea Centers for Disease Control and Prevention on various dates in 2018 (2018-01-03-C-A, 2018-01-03-P-A, 2018-01-03-2C-A, 2018-01-03-3C-A).

### Assessment of soda consumption

The intake of soda was evaluated using a 24-h dietary recall technique, following the coding guidelines of the KNHANES^(21)^. We used the intake of sustenance classification, specifically the food code (NF-INTK). A total of thirty-five different types of carbonated beverages were categorised, including soda, lemonade, orange soda, grape soda, cola, tonic water, fruit-flavoured sodas and other carbonated drinks, excluding artificially sweetened or diet versions. In this study, the adult population was categorised into five groups based on their soda consumption levels. These five groups include one group for non-drinkers and four quartiles for soda consumers. The quartiles for soda consumption were defined as follows. For the overall adult population, the quartiles were defined as: Q1 < 53 g/d, Q2 53–< 210 g/d, Q3 210–< 373 g/d and Q4 ≥ 373 g/d. For men, the quartiles were: Q1 < 125 g/d, Q2 125–< 258 g/d, Q3 258–< 420 g/d and Q4 ≥ 420 g/d. For women, the quartiles were: Q1 < 42 g/d, Q2 42–< 189 g/d, Q3 189–< 315 g/d and Q4 ≥ 315 g/d.

### Assessment of metabolic syndrome

In order for a diagnosis of MetS to be made, a person must exhibit at least three of the following criteria (1) abdominal obesity (waist circumference ≥ 90 cm for men and ≥ 85 cm for women); (2) elevated blood pressure (systolic blood pressure ≥ 130 mmHg or diastolic blood pressure ≥ 85 mmHg); (3) low HDL-cholesterol (fasting HDL-cholesterol < 40 mg/dl for men and < 50 mg/dl for women); (4) hypertriglyceridaemia (fasting triglyceride ≥ 150 mg/dl) and (5) hyperglycaemia (fasting plasma glucose ≥ 100 mg/dl). The researchers meticulously measured the waist circumferences of the participants, noting the measurements to the nearest 0·1 cm. Blood pressure readings were then taken after a brief 5-min rest in a seated position, with three readings recorded at 30-s intervals. The average of the second and third readings of both systolic and diastolic pressure was utilised for further examination. Fasting blood samples were collected after ≥ 8 h of fasting to measure HDL-cholesterol, triglycerides and fasting plasma glucose. Lipid profiles were analysed using the enzymatic method with an automatic biochemical analyser (Hitachi 7600-210 in 2017–2018; Labospect 008AS in 2019–2021). HDL-cholesterol was measured using a homogenous enzymatic colourimetric method in 2017–2018 and an enzymatic method in 2019–2021. Triglycerides, HDL-cholesterol and fasting plasma glucose levels were assessed in accordance with the protocols outlined by the National Cholesterol Education Program Adult Treatment Panel III^(22)^ and the Korean Society for the Study of Obesity criteria^(23)^.

### Confounding variables

Information regarding demographic and lifestyle variables, encompassing age, diet quality, physical activity levels and alcohol consumption, was gathered through personal interviews or self-administered questionnaires. Educational attainment was divided into three categories: ‘middle school or below’, ‘high school’ and ‘college or higher’. Alcohol consumption was stratified into the following categories: never/rarely, 1–4 times per month and ≥ 2 times per week. We categorised physical activity into high and low levels. High physical activity was defined as participating in at least 150 min of moderate-intensity activity per week, at least 75 min of vigorous-intensity activity per week, or a combination of both moderate and vigorous activities totalling at least 150 min per week (with 1 min of vigorous activity equating to 2 min of moderate activity). Diet quality was assessed as either high or low using a revised version of the Diet Quality Index for Koreans (DQI-K). The evaluation considered eight factors, including protein, cholesterol, whole grains, fruits, vegetables, sodium and the proportion of energy derived from fat and saturated fat. Each factor was allocated DQI-K points according to predefined thresholds, such as the Korean Dietary Reference Intakes. The total DQI-K score for each participant ranged from 0 to 9. A DQI-K score of 0–4 represented high diet quality, while a score of 5–9 represented low diet quality^(24,25)^. In addition, the total amount of energy consumed was meticulously calculated and examined as a continuous factor.

### Statistical analysis

We classified individuals into five groups based on soda consumption: non-drinkers, and four quartiles (Q1, Q2, Q3 and Q4) for soda consumers, for the total adult population. We utilised the PROC SURVEYREG and PROC SURVEYFREQ procedures to calculate the mean and prevalence of demographic and lifestyle variables using SAS 9.4 software. Multivariable-adjusted OR and 95 % CI for MetS according to soda consumption were determined with the PROC SURVEYLOGIST procedure. We conducted a stratified analysis based on the COVID-19 pandemic, dividing data into pre-COVID-19 (2017–2019) and post-COVID-19 (2020–2021) periods. The interaction between soda consumption and the COVID-19 periods in relation to MetS was analysed using PROC SURVEYLOGISTIC. All statistical analyses were performed with SAS 9.4 software. A two-tailed *P*-value of less than 0·05 was considered statistically significant.

## Results

### General characteristics

Table 1 shows the characteristics of Korean adults by soda consumption. Compared with people in the lowest group (non-drinkers), those in the highest group (Q4) of soda consumption were more likely to be younger. For men, there was a significant association between educational attainment and soda consumption. Individuals in the highest consumption category (Q4) were less likely to have attained a college education or higher. Both men and women in the high soda consumption group tended to consume less alcohol compared with those who did not consume soda. Additionally, a higher prevalence of current smoking was observed among women in this group. Physical activity levels did not show significant differences across the various soda consumption groups. Diet quality, evaluated through the DQI-K, indicated that individuals with high soda consumption had higher DQI-K scores, reflecting poorer dietary quality. These individuals also had higher total energy intakes, with a larger proportion of their caloric intake derived from carbohydrates and lower proportions from proteins.

**Table 1.** Characteristics of study population according to soda consumption in Korean adults aged 19–64 years* Table 1 long description.

	Soda consumption										*P* value^§^
	Non-drinkers		Q1		Q2		Q3		Q4		
	Mean	se	Mean	se	Mean	se	Mean	se	Mean	se	
Men, *n*	4174		328		343		370		297		
Age (years)	40·7	0·2	39·7	0·7	37·4	0·6	33·8	0·5	30·4	0·5	<0·0001
Body mass index (kg/m^2^)	24·6	0·1	25·1	0·2	25·0	0·2	24·6	0·2	25·3	0·3	0·016
	*n*	%	*n*	%	*n*	%	*n*	%	*n*	%	
Education, *n* (%)											0·035
≤ Middle school	357	6·8	17	3·8	14	2·7	15	2·9	7	1·7	
High school	1504	39·1	113	35·3	136	43·4	153	43·3	146	51·5	
≥ College	2128	54·1	187	60·9	181	54·0	188	53·9	137	46·8	
Covid, *n* (%)											0·058
2017–2019	2754	61·5	227	64·3	212	55·4	230	55·6	183	57·0	
2020–2021	1420	38·5	101	35·7	131	44·4	140	44·4	114	43·0	
Alcohol consumption, *n* (%)											0·0002
Never/rarely	1112	27·4	83	25·2	102	30·2	114	30·7	94	31·1	
1–4 times/month	1684	41·0	114	36·3	146	43·9	163	44·6	142	49·4	
≥ 2 times/week	1362	31·6	130	38·5	94	25·9	92	24·7	60	19·5	
Smoking, *n* (%)											0·444
Non-smoker	1203	31·1	89	28·7	104	31·1	130	36·1	123	42·0	
Former smoker	1477	34·3	119	35·3	109	31·9	91	25·7	69	21·9	
Current smoker	1476	34·6	119	36·0	129	37·0	148	38·3	104	36·1	
Physical activity^†^, *n* (%)											0·918
Low	1531	47·4	127	48·5	129	46·3	130	46·7	106	44·2	
High	1602	52·6	116	51·5	143	53·7	148	53·3	130	55·8	
DQI-K, *n* (%)											<0·0001
High diet quality	3390	79·4	250	79·9	229	65·2	239	62·0	175	59·0	
Low diet quality	784	20·6	78	24·1	114	34·8	131	38·0	122	41·0	
	Mean	se	Mean	se	Mean	se	Mean	se	Mean	se	
Total energy intake^‡^ (kcal)	1483·2	64·6	1867·4	78·5	1614·2	83·1	1667·6	78·1	1824·2	45·6	<0·0001
Percent from energy^‡^ (%)											
Carbohydrate	65·9	0·7	64·4	1·0	64·8	1·0	68·1	0·9	69·3	0·7	0·005
Protein	16·1	0·3	16·2	0·4	16·2	0·4	15·5	0·4	14·6	0·3	0·022
Fat	24·3	0·6	23·1	0·7	25·3	0·8	24·8	0·7	23·6	0·5	<0·0001
Women, *n*	6215		331		348		329		316		
Age (years)	41·3	0·2	41·2	0·7	36·2	0·7	34·5	0·7	31·6	0·6	<0·0001
Body mass index (kg/m^2^)	22·8	0·1	22·8	0·2	22·5	0·2	22·3	0·2	22·8	0·3	0·379
	*n*	%	*n*	%	*n*	%	*n*	%	*n*	%	
Education, *n* (%)											0·148
≤ Middle school	681	8·7	18	5·2	21	4·6	19	3·8	10	2·4	
High school	2181	37·0	125	37·5	125	40·7	124	42·1	129	44·0	
≥ College	3136	54·3	180	57·3	191	54·7	173	54·1	166	53·6	
Covid, *n* (%)											0·258
2017–2019	4076	60·1	229	62·2	245	65·3	217	59·5	189	55·5	
2020–2021	2139	39·9	102	37·8	103	34·7	112	40·5	127	44·5	
Alcohol consumption, *n* (%)											0·023
Never/rarely	3077	47·9	153	44·2	160	45·3	139	42·0	125	36·7	
1–4 times/month	2253	37·8	127	40·2	138	40·1	142	43·3	137	46·6	
≥ 2 times/week	859	14·2	51	15·6	50	14·6	47	14·8	53	16·7	
Smoking, *n* (%)											0·001
Non-smoker	5397	70·5	281	85·1	288	80·7	274	83·7	244	75·2	
Former smoker	457	6·1	33	10·1	31	10·9	33	9·4	32	11·0	
Current smoker	332	4·9	16	4·8	29	8·4	21	6·9	39	13·8	
Physical activity^†^, *n* (%)											0·708
Low	2576	53·1	143	51·8	156	56·1	131	49·1	133	53·4	
High	2112	46·9	123	48·2	100	43·9	121	50·9	119	46·6	
DQI-K, *n* (%)											<0·0001
High diet quality	5339	84·7	275	81·1	251	70·9	221	63·0	205	64·1	
Low diet quality	876	15·3	56	18·9	97	29·1	108	37·0	111	35·9	
	Mean	se	Mean	se	Mean	se	Mean	se	Mean	se	
Total energy intake^‡^ (kcal)	1145·3	52·2	1394·0	62·8	1246·7	70·0	1325·7	65·6	1434·6	41·1	<0·0001
Percent from energy^‡^ (%)											
Carbohydrate	66·2	0·7	64·7	1·0	64·7	1·2	66·0	1·0	68·4	0·7	<0·0001
Protein	13·4	0·3	13·3	0·4	13·0	0·4	12·6	0·4	11·8	0·3	0·018
Fat	23·4	0·6	23·3	0·8	24·7	0·9	23·9	0·8	23·9	0·6	<0·0001

DQI-K, a modified diet quality index for Koreans; *Values are presented as means (standard errors) for continuous variables, and number and weighted % for categorical variables; ^†^High physical activity was defined as at least 75 min of vigorous activity per week, at least 150 min of moderate activity per week or at least 150 min of a combination of vigorous and moderate activity per week; ^‡^Adjusted for age (continuous), education (≤ middle school, high school, or ≥ college), DQI-K (high diet quality, low diet quality), alcohol consumption (never/rarely, 1–4/month, or ≥ 2/week), smoking status (non-smoker, former smoker or current smoker), physical activity (low or high) and total energy intake (continuous). ^§^ *P*-values obtained from the χ2 test for categorical variables and from PROC SURVEYREG procedure for continuous variables.

DQI-K, Diet Quality Index for Koreans.

### Soda consumption and metabolic syndrome

Table 2 shows the results of multivariable logistic regression analysis examining the association between soda consumption and MetS and its components. After adjusting for multiple covariates, there was no significant association between soda consumption and MetS overall. For the components of MetS, however, adults in the highest quartile of soda consumption (≥ 373 g/d) had 37 % higher odds of abdominal obesity compared with non-drinkers (OR = 1·37, 95 % CI: 1·09, 1·73; *P*-trend = 0·006). Similarly, higher soda consumption was associated with an increased odd of hypertriglyceridaemia, with individuals in Q3 (210–< 373 g/d) and Q4 (≥ 373 g/d) having 62 % (OR = 1·62, 95 % CI: 1·23, 2·12) and 29 % higher odds (OR = 1·29, 95 % CI: 1·00, 1·67), respectively, compared with non-drinkers (*P*-trend = 0·003).

**Table 2.** Multivariable adjusted OR for metabolic syndrome according to soda consumption and types in Korean adults aged 19–64 years Table 2 long description.

	Soda consumption									*P* for trend
		Q1		Q2		Q3		Q4		
	Non-drinkers	OR	95 % CI	OR	95 % CI	OR	95 % CI	OR	95 % CI	
All adults (2290 cases/13 051 subjects)										
No. of cases/subjects	1831/10 389	131/660		110/765		107/586		111/651		
Median intake (g/d)	0	7·5		169·6		304·8		607·3		
Metabolic syndrome										
Model 1*	1·0 (reference)	1·30	1·05, 1·62	0·99	0·79, 1·26	1·39	1·08, 1·80	1·40	1·09, 1·78	0·002
Model 2^†^	1·0 (reference)	1·20	0·92, 1·56	0·89	0·68, 1·18	1·21	0·90, 1·62	1·18	0·89, 1·58	0·233
MetS components^†^										
Abdominal obesity (Men: ≥ 90 cm, Women: ≥ 85 cm)	1·0 (reference)	1·12	0·88, 1·42	1·14	0·92, 1·42	1·11	0·87, 1·42	1·37	1·09, 1·73	0·006
Hypertriglyceridaemia (≥ 150 mg/dl)	1·0 (reference)	1·06	0·83, 1·36	1·12	0·89, 1·42	1·62	1·23, 2·12	1·29	1·00, 1·67	0·003
Low HDL-cholesterol (Men: ≤ 40 mg/dl, Women: ≤ 50 mg/dl)	1·0 (reference)	0·91	0·70, 1·18	0·97	0·77, 1·23	1·32	1·01, 1·71	1·06	0·83, 1·35	0·262
Elevated blood pressure (SBP, systolic blood pressure: ≥ 130 mmHg, DBP, diastolic blood pressure: ≥ 85 mmHg)	1·0 (reference)	1·32	1·03, 1·69	0·97	0·74, 1·27	1·22	0·92, 1·64	1·03	0·76, 1·38	0·705
Hyperglycaemia (≥ 100 mg/dl)	1·0 (reference)	0·96	0·74, 1·25	0·82	0·65, 1·04	1·19	0·91, 1·56	1·19	0·91, 1·54	0·187
Men (1413 cases/5512 subjects)										
No. of cases/subjects	1084/4174	99/328		87/343		85/370		58/297		
Median intake (g/d)	0	18·5		200·2		356·0		710·4		
Metabolic Syndrome										
Model 1^‡^	1·0 (reference)	1·34	1·03, 1·76	1·16	0·88, 1·52	1·11	1·82, 1·50	1·07	0·77, 1·49	0·519
Model 2^§^	1·0 (reference)	1·27	0·92, 1·76	1·05	0·77, 1·44	1·14	0·82, 1·60	1·05	0·71, 1·54	0·669
MetS components^§^										
Abdominal obesity (Men: ≥ 90 cm)	1·0 (reference)	1·18	0·88, 1·58	1·25	0·95, 1·66	0·99	0·73, 1·33	1·32	0·96, 1·81	0·117
Hypertriglyceridaemia (≥ 150 mg/dl)	1·0 (reference)	1·00	0·73, 1·37	1·07	0·80, 1·44	1·50	1·11, 2·02	1·14	0·83, 1·57	0·072
Low HDL-cholesterol (Men: ≤ 40 mg/dl)	1·0 (reference)	1·06	0·73, 1·54	1·20	0·87, 1·65	1·30	0·94, 1·81	0·78	0·51, 1·18	0·816
Elevated blood pressure (SBP: ≥ 130 mmHg, DBP: ≥ 85 mmHg)	1·0 (reference)	1·22	0·89, 1·68	1·15	0·83, 1·61	1·26	0·91, 1·74	0·91	0·61, 1·35	0·860
Hyperglycaemia (≥ 100 mg/dl)	1·0 (reference)	1·09	0·81, 1·48	0·95	0·69, 1·30	1·17	0·85, 1·61	1·24	0·86, 1·78	0·214
Women (877 cases/7539 subjects)										
No. of cases/subjects	747/6215	43/331		24/348		28/329		35/316		
Median intake (g/d)	0	4·3		132·6		238·9		511·0		
Metabolic syndrome										
Model 1^‡^	1·0 (reference)	1·17	0·81, 1·70	0·79	0·48, 1·30	0·65	0·41, 1·03	1·59	1·04, 2·44	0·259
Model 2^§^	1·0 (reference)	1·18	0·78, 1·80	0·68	0·37, 1·28	0·69	0·41, 1·16	1·70	1·08, 2·68	0·154
MetS components^§^										
Abdominal obesity (Women: ≥ 85 cm)	1·0 (reference)	1·09	0·74, 1·59	1·00	0·66, 1·52	1·04	0·70, 1·52	1·63	1·12, 2·38	0·018
Hypertriglyceridaemia (≥ 150 mg/dl)	1·0 (reference)	1·37	0·96, 1·94	0·98	0·64, 1·51	1·62	1·09, 2·41	1·83	1·23, 2·74	0·001
Low HDL-cholesterol (Women: ≤ 50 mg/dl)	1·0 (reference)	0·85	0·61, 1·17	0·85	0·60, 1·21	0·97	0·69, 1·37	1·46	1·06, 2·01	0·043
Elevated blood pressure (SBP: ≥ 130 mmHg, DBP: ≥ 85 mmHg)	1·0 (reference)	1·40	0·99, 1·98	0·96	0·58, 1·59	0·47	0·26, 0·85	1·29	0·76, 2·20	0·982
Hyperglycaemia (≥ 100 mg/dl)	1·0 (reference)	0·72	0·47, 1·10	0·79	0·52, 1·19	0·76	0·50, 1·14	1·41	0·97, 2·07	0·268

*Adjusted for age (continuous) and sex; ^†^Adjusted for age (continuous), sex, education (≤ middle school, high school, or ≥ college), KDQI (high diet quality, low diet quality), alcohol consumption (never/rarely, 1–4/month, or ≥ 2/week), smoking status (non-smoker, former smoker or current smoker), physical activity (low or high) and total energy intake (continuous); ^‡^Adjusted for age (continuous); ^§^Adjusted for age (continuous), education (≤ middle school, high school, or ≥ college), DQI-K (high diet quality, low diet quality), alcohol consumption (never/rarely, 1–4/month, or ≥ 2/week), smoking status (non-smoker, former smoker or current smoker), physical activity (low or high) and total energy intake (continuous).

MetS, metabolic syndrome.

When analysed by gender, women in the highest quartile of soda consumption (≥ 315 g/d) had 70 % higher odds of having MetS compared with non-drinkers (OR = 1·70, 95 % CI: 1·08, 2·68). Additionally, women in Q4 exhibited 63 % higher odds of abdominal obesity (OR = 1·63, 95 % CI: 1·12, 2·38, *P*-trend = 0·018) and 46 % higher odds of low HDL cholesterol (OR = 1·46, 95 % CI: 1·06, 2·01, *P*-trend *=* 0·043). For hypertriglyceridaemia, soda consumption in Q3 (189–< 315 g/d) and Q4 (≥ 315 g/d) was associated with 62 % (OR = 1·62, 95 % CI: 1·09, 2·41) and 83 % (OR = 1·83, 95 % CI: 1·23, 2·74) higher odds (*P*-trend = 0·001). Notably, these associations were not observed in men.

Further analysis considered the potential impact of the COVID-19 pandemic on the relationship between soda consumption and MetS. Table 3 presents these findings. During the post-pandemic period, adults in the highest quartile of soda consumption (≥ 416 g/d) showed a significantly higher odds of MetS compared with non-drinkers (OR = 1·56, 95 % CI: 1·04, 2·34), while the association was not significant in the pre-pandemic period (*P*–interaction = 0·031). The observed interaction with COVID-19 pandemic, however, was not significant when analysed separately by gender.

**Table 3.** Multivariable adjusted OR for metabolic syndrome according to soda consumption by pre and post COVID-19 pandemic in Korean adults aged 19–64 years Table 3 long description.

Soda consumption	Covid^‡^								*P* for interaction
	Pre-pandemic				Post-pandemic				
	No. of cases/subjects		OR	95 % CI	No. of cases/ subjects		OR,	95 % CI	
	*n*	%			*n*	%			
Adults*									0·031
Non-drinkers	1211/6830	78·4	1·0 (reference)		620/3559	77·1	1·0 (reference)		
Q1	83/438	5·0	1·08	0·74, 1·60	50/232	5·1	1·31	0·92, 1·87	
Q2	53/438	5·2	0·59	0·38, 0·90	44/235	5·7	1·23	0·83, 1·83	
Q3	76/425	5·3	1·22	0·85, 1·75	44/237	6·1	1·03	0·69, 1·56	
Q4	60/431	6·2	0·88	0·59, 1·30	49/226	6·0	1·56	1·04, 2·34	
Men^†^									0·114
Non-drinkers	723/2754	75·4	1·0 (reference)		361/1420	72·5	1·0 (reference)		
Q1	56/208	5·8	1·07	0·65, 1·77	44/130	7·2	1·37	0·93, 2·03	
Q2	46/211	6·0	0·65	0·42, 1·00	36/116	6·5	1·39	0·87, 2·23	
Q3	48/218	6·1	1·24	0·77, 1·99	36/126	7·2	1·36	0·83, 2·23	
Q4	36/215	6·8	0·65	0·38, 1·11	27/114	6·6	1·46	0·87, 2·45	
Women^†^									0·549
Non-drinkers	488/4076	81·5	1·0 (reference)		259/2139	81·7	1·0 (reference)		
Q1	27/224	4·2	1·03	0·59, 1·78	15/111	4·2	1·20	0·64, 2·26	
Q2	15/218	4·4	0·66	0·29, 1·49	6/113	4·5	0·64	0·23, 1·74	
Q3	23/220	4·7	1·05	0·56, 1·98	9/115	5·1	0·78	0·36, 1·70	
Q4	21/218	5·2	1·65	0·93, 2·93	14/105	4·4	1·54	0·77, 3·08	

*Adjusted for covariates including, age (continuous), sex, education (≤ middle school, high school, or ≥ college), KDQI (high diet quality, low diet quality), alcohol consumption (never/rarely, 1–4/month or ≥ 2/week), smoking status (non-smoker, former smoker or current smoker), physical activity (low or high) and total energy intake (continuous); ^†^Adjusted for covariates including, age (continuous), education (≤ middle school, high school or ≥ college), DQI-K (high diet quality, low diet quality), alcohol consumption (never/rarely, 1–4/month, or ≥ 2/week), smoking status (non-smoker, former smoker or current smoker), physical activity (low or high) and total energy intake (continuous); ^‡^Participants were classified based on whether their data were collected before or after the COVID-19 pandemic.

## Discussion

Our study shows a significant association between soda consumption and the risk of MetS, particularly among women. Women in the highest quartile of soda consumption had a 70 % higher risk of MetS compared with non-drinkers, even after adjusting for potential confounders. Additionally, high soda consumption in women was significantly associated with specific MetS components, including abdominal obesity, low HDL-cholesterol and hypertriglyceridaemia. In contrast, no significant associations were observed between soda consumption and MetS or its components in men. It is important to note that men consumed larger amounts of soda overall, as reflected in the higher quartile cut-off values compared with women. Despite this higher level of consumption in men, the significant association with MetS was observed only among women. This finding suggests that women may be more susceptible to the adverse metabolic effects of soda consumption.

Our findings are consistent with previous studies that have highlighted the adverse metabolic effects of soda consumption, particularly among women. For example, Shin *et al.* reported a 61 % higher risk of MetS prevalence in Korean women aged 35–65 years, consuming ≥ 1 serving of SSB per day compared with women in the non-SSB-drinker group^(15)^. Similarly, Kang *et al.* found that Korean women aged 40–69 consuming ≥ 4 servings per week of soft drinks had an 82 % higher risk of MetS prevalence compared with non-drinkers^(26)^. These studies, along with findings by An *et al.* ^(27)^, consistently demonstrate gender specific risk patterns, supporting our results that adult women may be more vulnerable to the metabolic risks associated with soda consumption compared with men. Sex differences likely reflect complex interactions between hormones and fructose metabolism. Oestrogen influences the expression and activity of fructose-metabolising enzymes, including ketohexokinase-C, and lipogenic transcription factors such as carbohydrate response element-binding protein, creating metabolic environments where fructose preferentially undergoes de novo lipogenesis^(28,29)^. Under chronic fructose exposure, ketohexokinase-C activation appears to overwhelm oestrogen’s protective effects on endoplasmic reticulum stress pathways, with this relationship being particularly pronounced in females^(30)^. Women’s higher subcutaneous adipose tissue facilitates visceral fat redistribution under chronic fructose load, creating a ‘double-hit’ metabolic burden through both increased hepatic lipogenesis and enhanced peripheral fat storage^(31–33)^. This interplay between hormonal status and fructose metabolism may explain why women, despite consuming less soda overall, demonstrate stronger associations with MetS risk.

Beyond biological mechanisms, psychosocial factors may contribute to the sex-specific associations observed in this study. Women demonstrate higher susceptibility to stress-induced emotional eating behaviours, particularly involving increased consumption of high-energy, palatable foods, including soda^(34,35)^. The COVID-19 pandemic intensified psychological distress, with women experiencing significantly higher levels of anxiety, depression and emotional eating compared with men^(36,37)^. Previous studies have shown that psychological distress is directly associated with increased intake of SSB among women^(38)^, indicating that unmeasured psychological variables such as anxiety may have influenced dietary patterns during this period. The heightened psychological burden experienced by women during the pandemic, combined with their greater tendency to use food as a coping mechanism, may contribute to understanding the overall sex-specific vulnerability to soda-related metabolic risks observed in our study. These findings highlight the importance of considering psychosocial determinants in future longitudinal research examining dietary behaviours and metabolic health outcomes, particularly over extended pandemic periods.

Excessive soda consumption has been strongly linked to MetS risk through various mechanisms. Soda, sweetened with high-fructose corn syrup, promotes hepatic de novo lipogenesis, increasing plasma triglycerides and contributing to metabolic disturbances^(39–43)^. Frequent soda consumers also tend to have unbalanced dietary patterns, such as higher calorie and fat intake with lower dietary fibre, which exacerbate these effects^(44,45)^. These metabolic disruptions provide a biological basis for the observed associations between soda consumption and MetS risk in our study. The Framingham Heart Study found a significant association between SSB consumption and non-alcoholic fatty liver disease, which is consistent with our findings of soda’s association with MetS risk and its components, such as abdominal obesity, low HDL cholesterol and hypertriglyceridaemia^(46)^. Another study conducted in Taiwan showed significant associations between higher SSB consumption and increased MetS, including waist circumference, total cholesterol, LDL cholesterol and triglycerides, both in males and females. However, an important sex difference was observed with SSB consumption significantly associated with elevated blood pressure and fasting blood glucose only in females. This discrepancy may be partially explained by the influence of sex hormones, particularly oestrogen, on lipid metabolism and blood pressure regulation. Oestrogen is known to positively modulate the renin-angiotensin system, promote fat transport and increase triglyceride and lipoprotein levels. These hormonal effects could render women more sensitive to the metabolic risks associated with SSB consumption compared with men.^(47)^. Together, these studies emphasise the need for global public health strategies to mitigate the adverse metabolic effects of soda consumption.

During the COVID-19 pandemic, soda consumption increased, with individuals in the highest quartile of intake showing a 56 % higher risk of MetS compared with non-drinkers. The COVID-19 pandemic seems to further impact soda consumption and MetS prevalence. Our study observed an increased proportion of adults consuming high amounts of soda and sugar during the pandemic, with a corresponding rise in MetS prevalence. These findings underscore the need for public health strategies to mitigate the adverse metabolic effects of soda consumption, exacerbated by the pandemic^(48,49)^.

This study, despite utilising large-scale KNHANES data, has some limitations. First, the cross-sectional design prevents establishing causal relationships between soda intake and MetS. Additionally, the reliance on 24-h recall data, reflecting only the previous day’s intake, may not fully capture habitual dietary patterns. Furthermore, our study did not measure psychological variables such as anxiety, depression or stress levels, which may have influenced dietary behaviours during the pandemic period and contributed to the sex-specific associations observed. However, this study is the first to explore the association between soda consumption and MetS during the COVID-19 period in Korean adults, distinguishing soda from total SSB. Moreover, the large, nationally representative sample enhances generalizability, and the consistent use of 24-h recall data from 2017 to 2021 ensures accuracy. Therefore, this detailed methodology offers valuable insights into the relationship between soda intake and MetS, especially in the context of pandemic-related dietary changes.

In conclusion, this study reveals a significant association between soda consumption and an increased risk of MetS, particularly among Korean women, with findings observed during the COVID-19 pandemic, a period when both soda intake and MetS prevalence rose. While these findings provide valuable insights for informing public health policies and dietary recommendations, further longitudinal research is crucial to establish causal relationships and strengthen the evidence.

## Figure 1 Long description

The flowchart begins with 38,678 subjects who participated in the KNHANES 2017-2021. Of these, 31,365 completed the health interview, health examination, and nutrition survey. Subjects under 19 or over 65 years old (13,164) were excluded, leaving 18,201 participants. Further exclusions were made for individuals previously diagnosed with a stroke, myocardial infarction, or cancer, or taking medications for dyslipidemia, hypertension, or diabetes (including insulin) (3,981), pregnant or lactating women (188), those with extreme total energy intakes (274), individuals who fasted less than 8 hours (522), and those with missing MetS data (185). This resulted in a final eligible sample of 13,051 adults aged 19-64 years, divided into 5,512 men and 7,539 women.

Navigate back to Figure 1.

## Table 1 Long description

The table presents data on Korean adults aged 19 to 64 years, categorized by their soda consumption levels. It includes non-drinkers and four quartiles of soda consumption (Q1 to Q4). For men, the table shows age in years, body mass index in meters per square, education levels (less than middle school, high school, college), COVID-19 years (2020-2021 and 2020-2021), alcohol consumption (never/rarely, 1 to 4 times per month, 2 or more times per week), smoking status (non-smoker, former smoker, current smoker), physical activity levels (low, high), DQI-K scores (low diet quality, high diet quality), total energy intake in kilocalories, and the percentage of energy from carbohydrates, protein, and fat. Similar data is provided for women. Notable trends include younger age and lower educational attainment in higher soda consumption groups, less alcohol consumption, higher prevalence of smoking among women, and poorer diet quality with higher energy intake from carbohydrates and lower from proteins.

Navigate back to Table 1.

## Table 2 Long description

The table presents data on the association between soda consumption and metabolic syndrome (MetS) in Korean adults aged 19 to 64. It includes four quartiles of soda consumption (Q1, Q2, Q3, Q4) and non-drinkers, with corresponding odds ratios (OR) and 95% confidence intervals (CI) for MetS and its components. The table has 22 rows and 12 columns, detailing the number of cases and subjects, median intake in grams per day, and various MetS components such as abdominal obesity, hypertriglyceridaemia, low HDL-cholesterol, elevated blood pressure, and hyperglycaemia. Notable trends include higher odds of abdominal obesity and hypertriglyceridaemia in the highest quartile of soda consumption compared to non-drinkers.

Navigate back to Table 2.

## Table 3 Long description

The table presents data on the relationship between soda consumption and metabolic syndrome in Korean adults aged 19 to 64 years, comparing pre-pandemic and post-pandemic periods. The table is divided into three main groups: adults, men, and women. Each group is further divided into non-drinkers and quartiles of soda consumption (Q1, Q2, Q3, Q4). For each group and quartile, the table provides the number of cases and subjects, percentages, odds ratios (OR), and 95 percent confidence intervals (CI) for both pre-pandemic and post-pandemic periods. The table also includes P values for interaction. Notably, during the post-pandemic period, adults in the highest quartile of soda consumption (Q4) showed a significantly higher odds of metabolic syndrome compared with non-drinkers, with an OR of 1.56 and a 95 percent CI of 1.04 to 2.34. This association was not significant in the pre-pandemic period, with a P value for interaction of 0.031. The observed interaction with the COVID-19 pandemic was not significant when analyzed separately by gender.

Navigate back to Table 3.

## References

[1] Saklayen MG (2018) The global epidemic of the metabolic syndrome. Curr Hypertens Rep 20, 12. 29480368 10.1007/s11906-018-0812-z PMC5866840

[2] Alberti KGM & Zimmet P (2005) The metabolic syndrome – a new worldwide definition. Lancet 366, 1059–1062. 16182882 10.1016/S0140-6736(05)67402-8

[3] Johnson DW , Armstrong K , Campbell SB , et al. (2007) Metabolic syndrome in severe chronic kidney disease: prevalence, predictors, prognostic significance and effects of risk factor modification. Nephrology 12, 391–398. 17635756 10.1111/j.1440-1797.2007.00804.x

[4] Huang PL (2009) A comprehensive definition for metabolic syndrome. Dis Model Mech 2, 231–237. 19407331 10.1242/dmm.001180 PMC2675814

[5] Ranasinghe P , Mathangasinghe Y , Jayawardena R , et al. (2017) Prevalence and trends of metabolic syndrome among adults in the Asia-Pacific region: a systematic review. BMC Public Health 17, 101. 28109251 10.1186/s12889-017-4041-1 PMC5251315

[6] Cuevas A , Alvarez V & Carrasco F (2011) Epidemic of metabolic syndrome in Latin America. Curr Opin Endocrinol, Diabetes Obes 18, 134. 21358406 10.1097/MED.0b013e3283449167

[7] Lim S , Shin H , Song JH , et al. (2011) Increasing prevalence of metabolic syndrome in Korea: the Korean National Health and Nutrition Examination Survey for 1998–2007. Diabetes Care 34, 1323–1328. 21505206 10.2337/dc10-2109 PMC3114326

[8] Tran BT , Jeong BY & Oh OJ (2017) The prevalence trend of metabolic syndrome and its components and risk factors in Korean adults: results from the Korean National Health and Nutrition Examination Survey 2008–2013. BMC Public Health 17, 71. 28086850 10.1186/s12889-016-3936-6 PMC5237316

[9] Park D , Shin M-J , Després JP , et al. (2023) 20-year trends in metabolic syndrome among Korean adults from 2001 to 2020. JACC Asia 3, 491–502. 37396427 10.1016/j.jacasi.2023.02.007 PMC10308107

[10] Kim H , Kim S-E & Sung MK (2025) Sex and gender differences in obesity: biological, sociocultural, and clinical perspectives. World J Mens Health 43, 758–772. 40676890 10.5534/wjmh.250126 PMC12505483

[11] Duffey KJ & Popkin BM (2007) Shifts in patterns and consumption of beverages between 1965 and 2002. Obesity 15, 2739–2747. 18070765 10.1038/oby.2007.326

[12] Popkin BM (2010) Patterns of beverage use across the lifecycle. Physiol Behavior 100, 4–9. 10.1016/j.physbeh.2009.12.022 PMC2849916 20045423

[13] Lee H-S , Kwon S , Yon M , et al. (2014) Dietary total sugar intake of Koreans: based on the Korea National Health and Nutrition Examination Survey (KNHANES), 2008–2011. J Nutr Health 47, 268–276.

[14] Ministry of Food and Drug Safety (2024) Results of National Sodium and Sugar Intake Analysis. https://www.nifds.go.kr/index.do (accessed April 2024).

[15] Shin S , Kim S-A , Ha J , et al. (2018) Sugar-sweetened beverage consumption in relation to obesity and metabolic syndrome among Korean Adults: a cross-sectional study from the 2012–2016 Korean National Health and Nutrition Examination Survey (KNHANES). Nutrients 10, 1467. 30304842 10.3390/nu10101467 PMC6213560

[16] Anari R , Amani R & Veissi M (2019) Sugary beverages are associated with cardiovascular risk factors in diabetic patients. J Diabetes Metab Disord 18, 7–13. 31275869 10.1007/s40200-019-00383-5 PMC6582119

[17] Chun S , Choi Y , Chang Y , et al. (2016) Sugar-sweetened carbonated beverage consumption and coronary artery calcification in asymptomatic men and women. Am Heart J 177, 17–24. 27297845 10.1016/j.ahj.2016.03.018

[18] Cruz Neto J , Frota Cavalcante T & de Carvalho Félix ND (2022) Post-COVID-19 metabolic syndrome: a new challenge for nursing care. Invest Educ Enferm 41, e01. 10.17533/udea.iee.v41n1e01 PMC10152914 37071858

[19] Dissanayake H (2023) COVID-19 and metabolic syndrome. Best Pract Res Clin Endocrinol Metab 37, 101753. 36907785 10.1016/j.beem.2023.101753 PMC9977132

[20] Kweon S , Kim Y , Jang MJ , et al. (2014) Data resource profile: the Korea National Health and Nutrition Examination Survey (KNHANES). Int J Epidemiol 43, 69–77. 24585853 10.1093/ije/dyt228 PMC3937975

[21] Korea Centers for Disease Control and Prevention (2020) User Guide for the Eighth Korea National Health and Nutrition Examination Survey (KNHANES VIII); Cheongwon, Korea, 2020. https://knhanes.kdca.go.kr/knhanes/main.do (accessed April 2024).

[22] National Cholesterol Education Program (U S.) & Expert Panel on Detection, Evaluation, and Treatment of High Blood Cholesterol in Adults (2002) Third Report of the National Cholesterol Education Program (NCEP) Expert Panel on Detection, Evaluation, and Treatment of High Blood Cholesterol in Adults (Adult Treatment Panel III): Final Report. The Program. https://pubmed.ncbi.nlm.nih.gov/12485966/ (accessed April 2024).

[23] Kim B-Y , Kang SM , Kang JH , et al. (2021) 2020 Korean society for the study of obesity guidelines for the management of obesity in Korea. J Obes Metab Syndr 30, 81–92. 34045368 10.7570/jomes21022 PMC8277596

[24] Shim JE , Lee SY , Moon HK , et al. (2002) Comparative analysis and evaluation of dietary intake of Koreans by age groups: (4) the Korean diet quality index. J Nutr Health 35, 558–570.

[25] Lim J , Lee Y , Shin S , et al. (2018) An association between diet quality index for Koreans (DQI-K) and total mortality in Health Examinees Gem (HEXA-G) study. Nutr Res Pract 12, 258–264. 29854332 10.4162/nrp.2018.12.3.258 PMC5974072

[26] Kang Y & Kim J (2017) Soft drink consumption is associated with increased incidence of the metabolic syndrome only in women. Br J Nutr 117, 315–324. 28166856 10.1017/S0007114517000046

[27] An H-J , Kim Y & Seo YG (2023) Relationship between coffee, tea, and carbonated beverages and cardiovascular risk factors. Nutrients 15, 934. 36839290 10.3390/nu15040934 PMC9966641

[28] Softic S , Gupta MK , Wang GX , et al. (2017) Divergent effects of glucose and fructose on hepatic lipogenesis and insulin signaling. J Clin Invest 127, 4059–4074. 28972537 10.1172/JCI94585 PMC5663363

[29] Mirtschink P , Jang C , Arany Z , et al. (2018) Fructose metabolism, cardiometabolic risk, and the epidemic of coronary artery disease. Eur Heart J 39, 2497–2505. 29020416 10.1093/eurheartj/ehx518 PMC6037111

[30] Park S-H , Helsley RN , Fadhul T , et al. (2023) Fructose Induced KHK-C can increases ER Stress independent of its effect on lipogenesis to drive liver disease in diet induced and genetic models of NAFLD. Metabolism 145, 155591. 37230214 10.1016/j.metabol.2023.155591 PMC10752375

[31] Kovačević S , Brkljačić J , Vojnović Milutinović D , et al. (2021) Fructose induces visceral adipose tissue inflammation and insulin resistance even without development of obesity in adult female but not in male rats. Front Nutr 8, 749328. 34869524 10.3389/fnut.2021.749328 PMC8632624

[32] Stephenson EJ , Stayton AS , Sethuraman A , et al. (2022) Chronic intake of high dietary sucrose induces sexually dimorphic metabolic adaptations in mouse liver and adipose tissue. Nat Commun 13, 6062. 36229459 10.1038/s41467-022-33840-6 PMC9561177

[33] Chang E , Varghese M & Singer K (2018) Gender and sex differences in adipose tissue. Curr Diab Rep 18, 69. 30058013 10.1007/s11892-018-1031-3 PMC6525964

[34] Thompson SH & Romeo S (2015) Gender and racial differences in emotional eating, food addiction symptoms, and body weight satisfaction among undergraduates. JDO 2, 1–6.

[35] Finch LE & Tomiyama AJ (2015) Comfort eating, psychological stress, and depressive symptoms in young adult women. Appetite 95, 239–244. 26192221 10.1016/j.appet.2015.07.017

[36] Prowse R , Sherratt F , Abizaid A , et al. (2021) Coping with the COVID-19 pandemic: examining gender differences in stress and mental health among university students. Front Psychiatry 12, 650759. 33897499 10.3389/fpsyt.2021.650759 PMC8058407

[37] Güner Ö & Aydın A (2022) Determining the relationship between anxiety levels, stress coping styles, and emotional eating of women in the COVID-19 pandemic. Arch Psychiatr Nurs 41, 241–247. 36428056 10.1016/j.apnu.2022.08.002 PMC9385584

[38] Grieger JA , Habibi N , O’Reilly SL , et al. (2022) Psychological distress and its association with intake of sugar-sweetened beverages, discretionary foods, and alcohol in women during the COVID-19 pandemic in Australia. Nutrition 103–104, 111794. 36055124 10.1016/j.nut.2022.111794 PMC9427120

[39] David F (2005) Effect of fructose overfeeding and fish oil administration on hepatic de novo lipogenesis and insulin sensitivity in healthy men. Diabetes 54, 1907. 15983189 10.2337/diabetes.54.7.1907

[40] Diana IJ (2010) Increased fructose associates with elevated blood pressure: journal of the American Society of Nephrology. Clin Epidemiol J Am Soc Nephrol 21, 1543–1549. 10.1681/ASN.2009111111 PMC3013529 20595676

[41] Hwang I-S (1987) Fructose-induced insulin resistance and hypertension in rats. Hypertension 10, 512–516. 3311990 10.1161/01.hyp.10.5.512

[42] Angelis KD , Senador DD , Mostarda C , et al. (2012) Sympathetic overactivity precedes metabolic dysfunction in a fructose model of glucose intolerance in mice. Am J Physiology-Regulatory, Integrative Comp Physiol 302, R950–R957. 10.1152/ajpregu.00450.2011 PMC3330769 22319048

[43] Giacchetti G , Sechi LA , Griffin CA , et al. (2000) The tissue renin-angiotensin system in rats with fructose- induced hypertension: overexpression of type 1 angiotensin II receptor in adipose tissue. J Hypertens 18, 695. 10872553 10.1097/00004872-200018060-00006

[44] Duffey KJ & Popkin BM (2006) Adults with healthier dietary patterns have healthier beverage patterns. J Nutr 136, 2901–2907. 17056820 10.1093/jn/136.11.2901

[45] Park S , Kim DS & Kang S (2024) Gene–diet interactions in carbonated sugar-sweetened beverage consumption and metabolic syndrome risk: a machine learning analysis in a large hospital-based cohort. Clin Nutr ESPEN 64, 358–369. 39396701 10.1016/j.clnesp.2024.10.004

[46] Park WY , Yiannakou I , Petersen JM , et al. (2022) Sugar-sweetened beverage, diet soda, and nonalcoholic fatty liver disease over 6 years: the Framingham heart study. Clin Gastroenterol Hepatol 20, 2524–2532. 34752964 10.1016/j.cgh.2021.11.001 PMC9236136

[47] Kuo C-T , Chen D-R , Chan CC , et al. (2023) Sex differences in the association between sugar-sweetened beverages consumption and metabolic risks among the working-age population in Taiwan. Public Health Nutr 26, 653–660. 35851091 10.1017/S1368980022001549 PMC9989700

[48] Oh K , Park S , Park S , et al. (2021) Changes in food consumption and nutrient intakes in Korean adults before and during the COVID-19 pandemic: 2011–2020 Korea National Health and Nutrition Examination Survey. Br J Nutr 126, 757–766.33198840

[49] Kim J & Kye S (2023) Lifestyle and dietary changes related to weight gain in college students during the COVID-19 pandemic. J Nutr Health 56, 288–299.

